# Preliminary hemocompatibility assessment of an innovative material for blood contacting surfaces

**DOI:** 10.1007/s10856-021-06556-0

**Published:** 2021-07-27

**Authors:** Martina Todesco, Elena Pontara, Chunyan Cheng, Gino Gerosa, Vittorio Pengo, Andrea Bagno

**Affiliations:** 1 Department of Industrial Engineering, University of Padua, via Marzolo 9, 35131 Padova, Italy; 2 L.i.f.e.L.a.b. Program, Consorzio per la Ricerca Sanitaria (CORIS), Veneto Region, Via Giustiniani 2, 35128 Padova, Italy; 3 Department of Cardiac, Thoracic and Vascular Sciences, University of Padova, via Giustiniani 2, 35127 Padova, Italy

## Abstract

Over the years, several devices have been created (and the development of many others is currently in progress) to be in permanent contact with blood: mechanical circulatory supports represent an example thereof. The hemocompatibility of these devices largely depends on the chemical composition of blood-contacting components. In the present work, an innovative material (hybrid membrane) is proposed to fabricate the inner surfaces of a pulsatile ventricular chamber: it has been obtained by coupling a synthetic polymer (e.g., commercial polycarbonate urethane) with decellularized porcine pericardium. The hemocompatibility of the innovative material has been preliminarily assessed by measuring its capacity to promote thrombin generation and induce platelet activation. Our results demonstrated the blood compatibility of the proposed hybrid membrane.

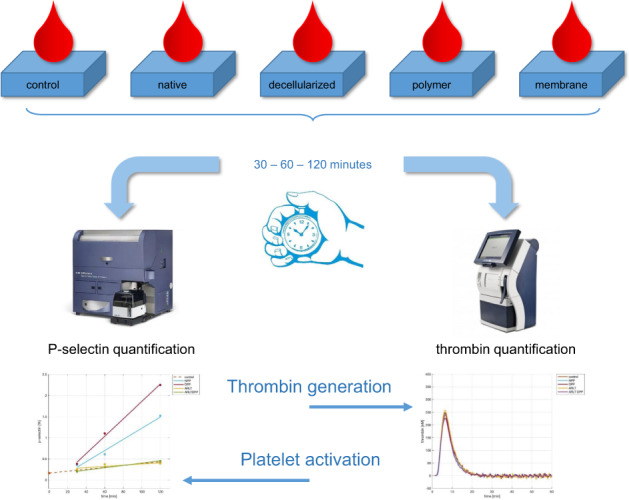

## Introduction

Recent technological advancements allowed expanding the clinical exploitation of several biomedical devices to be surgically implanted into the body; many of them have to stay in permanent contact with blood. Examples of these kinds of devices are Total Artificial Hearts (TAHs), Ventricular Assist Devices (VADs), vascular grafts, and prosthetic mechanical heart valves. They are different in terms of structural and functional complexity, but they have to share a common constraint, which is hemocompatibility. Indeed, hemocompatibility involves several issues: from the absence of hemolysis and platelet activation [1, 2], to the prevention of cellular component consumption and activation of coagulation pathways [3]. Blood–biomaterial interactions are very complex: they are regulated by mechanisms that control many events in sequence, some of which have not been fully understood. Anyway, clinical practice demonstrates that blood compatibility concerns can cause hemorrhages, hemolysis, thrombosis, and thromboembolism [4].

In the present work, the evaluation of an innovative membrane is illustrated: it is obtained by coupling a synthetic polymer (commercial polycarbonate urethane) with decellularized porcine pericardium. While synthetic polymer gives the membrane the necessary mechanical resistance, the decellularized tissue provides hemocompatibility. More interestingly, the decellularized tissue is prone to repopulation by circulating cells: it will result in a stable endothelialization in vivo, avoiding the limitations of usual chemical fixation with glutaraldehyde. This membrane is intended for the production of an original ventricular chamber with enhanced hemocompatibility.

Upon the first contact between blood and one surface, proteins’ adsorption takes place: this event is directed by competitive exchanges (Vroman effect [5]) that redistribute adsorbed proteins depending on their relative affinity for the given material and on their concentrations in the bulk [6]. Eventually, adsorbed proteins mediate the interactions with platelets and blood cells; therefore, proteins largely control the fate of the blood-contacting surface. In particular, adsorbed proteins are responsible for the activation of platelets and the generation of thrombin. This latter is the key enzyme that converts fibrinogen into fibrin, which is the final product of the coagulation cascade resulting in thrombus formation. Platelets also are involved in thrombus formation by aggregation [1]; their activity is primarily associated with the initiation of the coagulation cascade. Platelets are activated upon contact with any surface different from the healthy endothelium. In particular, the glycoproteic receptor complex Ib-IX-V on the platelet membrane identifies exposed sub-endothelial collagen and, by means of the von Willebrand factor (vWF), platelets bind to the blood vessel wall. Moreover, exposed collagen can bind directly to platelet GP Ia/IIa and GP VI receptors [2]. Another stimulus causing platelet activation is their exposure to circulating agonists: thrombin is one of them [7]. Therefore, the hemocompatibility of a given surface can be preliminarily assessed by measuring its capacity to promote thrombin generation and induce platelet activation.

The present work aims at evaluating the hemocompatibility of an innovative material that is obtained by coupling a synthetic polymer (a hemocompatible polycarbonate urethane) with a decellularized biological tissue (porcine pericardium). Blood compatibility of this material (hybrid membrane, DPP-ARLT) is compared with the control and with native (NPP) and decellularized (DPP) porcine pericardia, and with the polymer alone (ARLT).

## Materials and methods

### Biological tissue preparation

Biological tissue preparation is illustrated in detail in [8]. Briefly: fresh pericardia of healthy animals (Duroc pigs, 9–14-months old) were supplied from local abattoirs. Within 2 h after animal death, pericardium was dissected free from its attachment at the base of the heart around great vessels; it was cleaned by removing retrosternal fat and surrounding connective tissues. For the fabrication of the hybrid membrane, porcine pericardium was withdrawn from the anterior right ventricular region. Tissue was treated according to the TriCol procedure, which was proposed for decellularizing the aortic valves [9]. At the end of the decellularization procedure, pericardial samples were stored at 4 °C in antibiotic and antimycotic solution with 3% penicillin–streptomycin (Sigma-Aldrich, Saint Louis, MO, USA) and 0.25% Amphotericin B (Carlo Erba, Cornaredo, Italy).

### Polymeric and hybrid membranes fabrication

Polymer was supplied as 22% (w/v) solutions in N,N-dimethylacetamide. Polymeric membranes (ARLT) were produced by dispensing the polycarbonate urethane solution (ChronoFlex AR-LT, CF, AdvanSource Biomaterials, Wilmington, MA, US) into a customized 50 × 50 mm^2^ aluminum frame, then dried under vacuum for 24 h at 80 °C. After complete drying and cooling, polymeric membranes were carefully detached from the frame.

Hybrid membranes (DPP-ARLT) were produced by solution casting and solvent evaporation [8]. Decellularized tissue samples were placed on the serosa side and fastened into the 50 × 50 mm^2^ frame. Over the other side (fibrosa) a volume of 5 mL polycarbonate urethane solution was gently poured. Hybrid membranes were dried for 24 h at 40 °C into a vacuum oven under aspiration (Raypa, Barcelona, Spain).

### Blood preparation

Human blood samples were obtained from the antecubital vein of a healthy volunteer (female, 28 years old), who was not allowed to take any drug 10 days before blood withdrawal.

A blood volume of 60 mL was collected in Vacutainer tubes containing 3.2% sodium citrate solution.

Disks of 8 mm diameter obtained from the investigated materials (NPP, DPP, ARLT, and DPP-ARLT) were placed in a polystyrene 48-well flat-bottom plate (Sarstedt, Nümbrecht, Germany). A blood volume of 500 μL was pipetted in each well. Polystyrene was assumed as positive control.

Multiwell plate was kept at room temperature in gentle agitations. After 30, 60, and 120 min a sample of blood was collected from each well.

### Platelet activation assessment

Platelet activation was measured by the expression of P-selectin on platelet surface through flow cytometry. Briefly, within 2 h after blood sampling, whole blood was incubated with the investigated materials. After 30, 60, and 120 min, five microliters of mixture was diluted in 45 μL HBS buffer (0.01 M Hepes pH 7.4, 0.14 M NaCl, and 2.5 mM CaCl_2_ solution), and incubated with titered antibodies CD41a (PE) for platelet identification and CD62p(APC) for P-selectin identification for 15 min in the dark. One milliliter of HBS buffer was added before acquisition. P-selectin positivity was calculated as percentage between CD41a(PE) and CD62p(APC) populations. Samples were acquired by Cyflow^®^ Space cytometer and analyzed by Partec FloMaxTM software (Partec, Münster, Germany). 20,000 events in platelet gate were acquired with a slow flow rate.

### Thrombin generation assay

Plasma of the volunteer was obtained by centrifuging twice blood at 12000 rpm for 10 min to remove cellular components.

TG was investigated as described by Hemker et al. [10]. In 96-well round bottom plate (Thermo Fisher Scientific, Waltham, Massachusetts, United States) a volume of 20 μL of PPP reagent (tissue factor 5 pmol/L and phospholipids 4 μmol/L) was added to one well and 20 μL of calibrator in another. Both reagents were supplied by Diagnostica Stago (Gennevilliers, France). A volume of 80 μL of plasma was added to both wells. Samples were assessed using the Calibrated Automated Thrombogram (CAT, Thrombinoscope BV, Stago, UK). TG was initiated by the addition of the fluorogenic substrate and calcium (Fluka-kit reagent, Diagnostica Stago) at zero time. Fluorescents were recorded for 60 min and convert to TG curves by Thrombinoscope software.

### Data analysis

When applicable, significant differences were assessed by the Kruskal–Wallis test (*p* value < 0.05) performed with Prism 8 (GraphPad Software, San Diego, CA, USA).

## Results

### Platelet activation

**Fig. 1:**
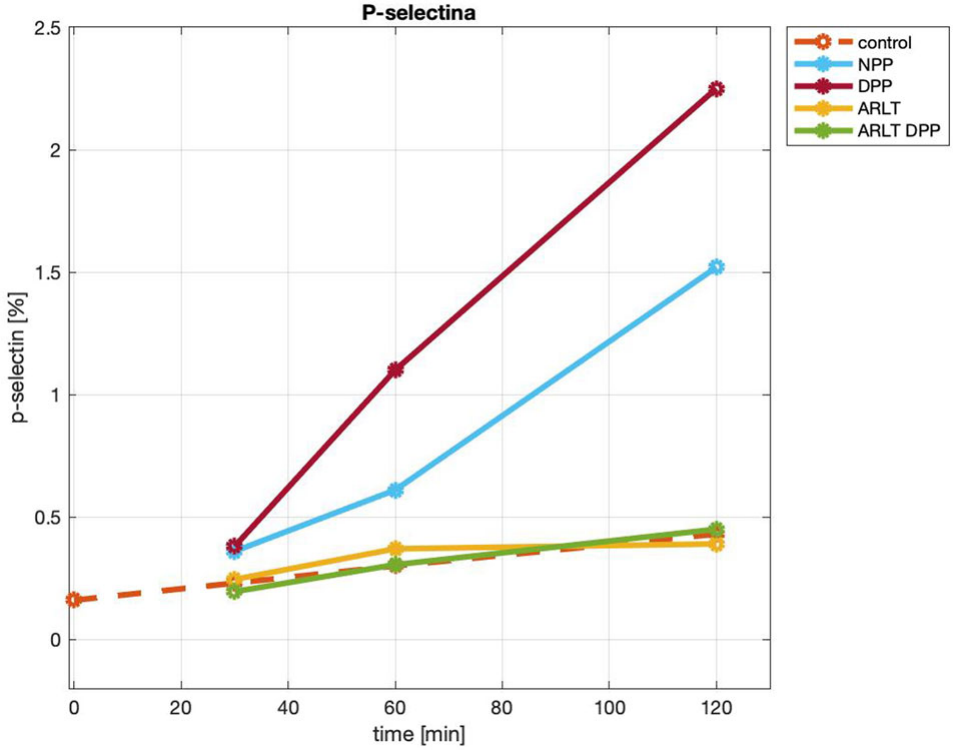
Values of platelet P-selectin [%] after 30, 60 and 120 minutes of blood incubation over the investigated surfaces

Platelet activation was assessed by measuring the amount of P-selectin expressed by platelets after 30, 60, and 120 min of blood incubation in contact with the investigated materials. Figure 1 illustrates the curves obtained by interpolating the different values measured at different time points. Two distinct trends are clearly identified. On the one hand, NPP and DPP, which is the biological tissue before and after decellularization, cause a fast increase of P-selectin in %: it goes from 0.36% (NPP) and 0.38% (DPP) at 30 min up to 1.52% and 2.25% at 120 min, respectively. On the other hand, control, polymer (ARLT) and hybrid membrane (DPP-ARLT) show values from 0.16% (control at time 0) up to a maximum of 0.45% (DPP-ARLT), with a slight increase over time. Moreover, it is worthy to compare the slope of the curves in Fig. 1: it can be considered as an index of the capacity to induce platelet activation. NPP and DPP have a slope of 1.32 and 2.05, respectively: these values are much higher than control (0.22), ARLT (0.14), and DPP-ARLT (0.28).

### Thrombin generation

**Fig. 2:**
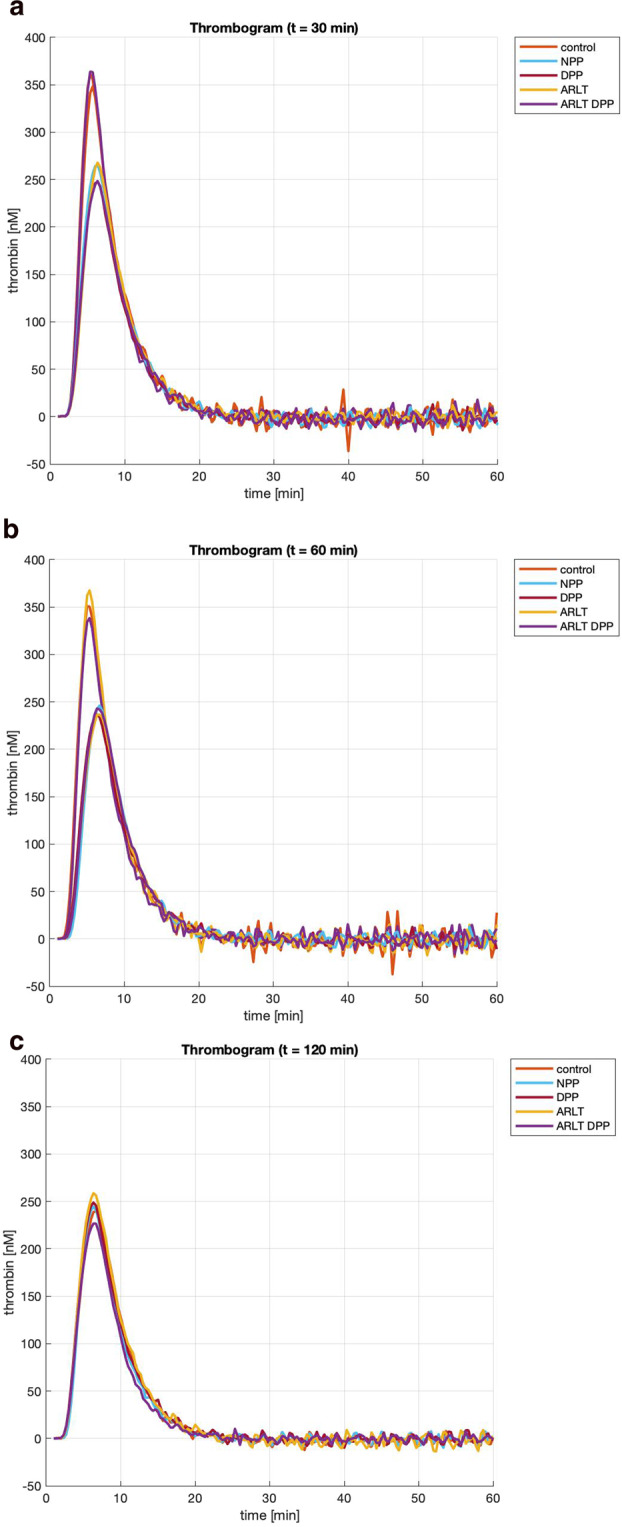
Thrombin generation curves collected after 30 (**A**), 60 (**B**) and 120 (**C**) minutes of blood incubation over the investigated surfaces. Thrombin concentration is expressed in nM

**Fig. 3:**
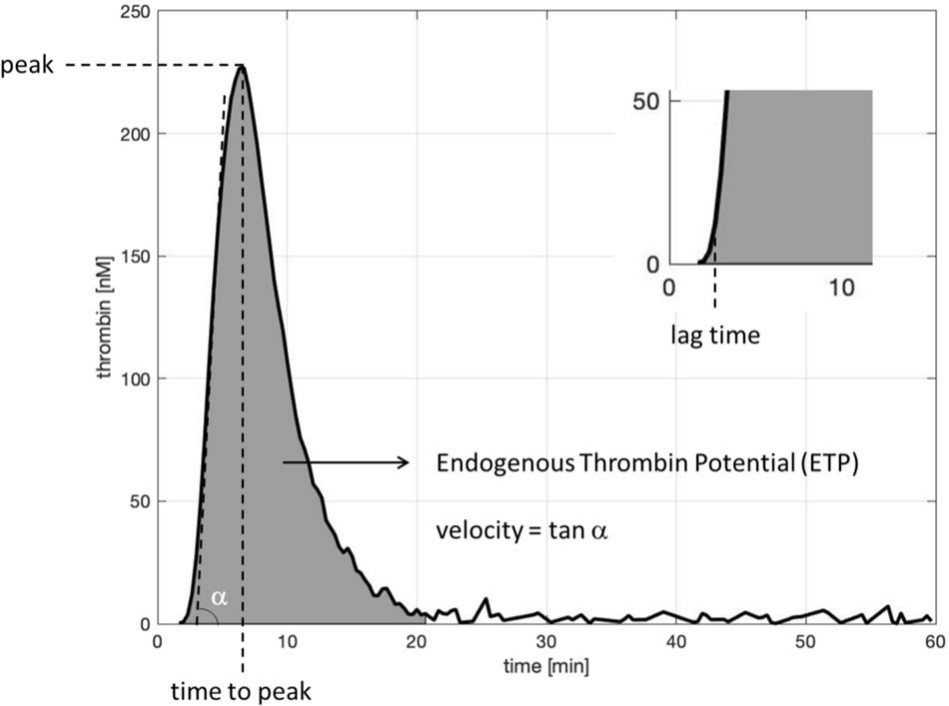
Schematic representation of thrombogram generated by the CAT method and the relative parameters of interest (lag time, peak, time to peak, endogenous thrombin potential and velocity index)

Three families of thrombin generation curves were obtained, after 30, 60, and 120 min of blood incubation in contact with the investigated materials (Fig. 2). At each time point, the curves corresponding to the different materials are largely overlapped. Generally, these curves show an initial increase of thrombin concentration, until reaching the maximum value; then, the curves go rapidly down to the baseline. From the experimental curves, five parameters are calculated (Fig. 3): their values are summarized in Tables from 1 to 5.

With regard to the parameter “peak” (Table 1), which identifies the maximum value reached by thrombin concentration, all materials exhibit values that are lower than the control at 0 min, with the exception of ARLT after 30 min. Over time, differences among the investigated materials are not significant (*p* value = 0.4): SD values decrease from 45.33 at time 30 to 11.27 at time 120.

**Table 1:** Peak values measured at different time points over the investigated surfaces

Time	control	NPP	DPP	ARLT	DPP-ARLT	Mean	SD
0	312,03						
30	349,01	266,98	243,9	339,49	305,00	300,87	45,33
60	299,22	244,48	236,51	301,72	290,4	274,46	31,42
120	239,83	243,90	248,55	257,71	227,15	243,43	11,27

Mean values and standard deviations (SD) are reported. Values are in [nM]

Differences in “lag time” values (Table 2) are not significant (*p* value = 0.0577). In this case, SD values vary from 0.13 (at 30 min) to 0.14 (at 120 min). Similarly, there are no significant differences in “time-to-peak” values (Table 3) (*p* value = 0.5755). In particular, SD values decrease from 30 min (0.35) to 120 min (0.17).

**Table 2:** Lagtime values measured at different time-points over the investigated surfaces

Time	control	NPP	DPP	ARLT	DPP-ARLT	Mean	SD
0	2,67					2,97	
30	2,83	3,17	3,00	2,92	2,92	3,00	0,13
60	3,00	3,33	3,00	2,84	2,84	3,13	0,20
120	3,00	3,33	3,17	3,00	3,17	2,97	0,14

Mean values and standard deviations (SD) are reported. Values are in [min]

**Table 3:** Ttpeak values measured at different time-points over the investigated surfaces

Time	control	NPP	DPP	ARLT	DPP-ARLT	Mean	SD
0	5,50						
30	5,50	6,33	6,33	5,83	5,92	5,98	0,35
60	6,00	6,67	6,33	6,00	6,00	6,20	0,30
120	6,67	6,33	6,50	6,33	6,67	6,50	0,17

Mean values and standard deviations (SD) are reported. Values are in [min]

The parameter “velocity” (Table 4), which represents the slope of the thrombin generation curve, reaches its maximum value for the control after 30 min: all other materials show similar values (*p* value = 0.5584): SD values decrease over time (from 23.34 at 30 min to 7.25 at 120 min).

**Table 4:** Velocity index values measured at different time-points over the investigated surfaces

Time	control	NPP	DPP	ARLT	DPP-ARLT	Mean	SD
0	110,73						
30	133,39	84,62	74,30	112,52	105,67	102,10	23,34
60	102,93	73,34	70,95	101,02	96,48	88,94	15,53
120	65,41	81,30	74,56	77,31	65,06	72,73	7,25

Mean values and standard deviations (SD) are reported. Values are in [nM/min]

The parameter “ETP” is calculated as the area of the peak (Table 5): it represents the overall amount of thrombin. The highest values are obtained for the control (at 0 and 30 min), whereas all other materials show lower values, whose SD decreases over time (from 146 at 30 min to 103 at 120 min). No significant difference is detectable (*p* value = 0.2).

**Table 5:** ETP (Endogen Thrombin Potential) values at different time-points over the investigated surfaces

Time	control	NPP	DPP	ARLT	DPP-ARLT	Mean	SD
0	2002,53						
30	2025,65	1772,74	1650,45	1942,76	1840,68	1846,46	146,00
60	1939,65	1649,39	1665,69	1862,46	1820,93	1787,62	126,29
120	1652,45	1585,16	1697,23	1759,20	1492,26	1637,26	103,00

Mean values and standard deviations (SD) are reported. Values are in [nM × min]

## Discussion

The present work aims at characterizing the hemocompatibility of an innovative material (hybrid membrane) intended for cardiovascular applications, compared with other materials. To this end, the capacity to promote platelet activation and induce thrombin generation was assessed. This kind of assessment is only one part of the overall process resulting in biocompatibility evaluation.

Anyway, the adoption of hemocompatible materials does not necessarily result in blood compatibility of the entire device: it also depends on structural [11] and chemical properties [12, 13], biological behavior of the interfacing surfaces, flow dynamics [14], and—last but not least—the overall design. A paper discussing the hemocompatibility issue for TAHs has been recently published [15]: it presents a detailed analysis of the pathophysiological events that may arise in currently developed TAHs and illustrates bioengineering solutions to prevent them.

The only surface fully compatible with blood is the healthy endothelium lining the lumen of blood vessels. Thus, the formation of a pseudo-neointima on blood-contacting surfaces can improve hemocompatibility by reducing the potential to cause thromboembolic complications [16, 17].

Platelet activation is a sensitive tool to test blood-compatibility of synthetic materials [18, 19]. Platelet activation can be triggered upon contact with any surface different from the healthy endothelium: both adhesion and consequent activation have been measured by microscopy [20, 21]. In other studies, hemocompatibility was investigated by assessing platelet adhesion and complement activation [22]. In the present work, platelet activation was estimated by measuring the expression of P-selectin after human blood incubation with different materials, including the one intended for cardiovascular applications. As to platelet activation, the investigated materials exhibited two different behaviors. Two of them, namely porcine pericardium before (NPP) and after decellularization (DPP), induced a rapid increase of P-selectin: this demonstrates that decellularization did not reduce rather increased the thrombogenic potential of the biological tissue. It is easy to speculate that cell removal due to decellularization resulted in the exposition of collagen fibers. Overall, these data confirm that, despite decellularization, the biological tissue is still thrombogenic. Other materials, namely control surface, synthetic polymer (ARLT), and hybrid membrane (DPP-ARLT) behave similarly: they did not promote P-selectin expression over time. Therefore, the presence of the synthetic polymer abrogates the thrombogenic potential of the decellularized tissue.

Thrombin generation assay measures the ability of a plasma sample to generate thrombin following in vitro activation of coagulation. Thrombin generation curves, which are characterized by a lag phase followed by the formation and subsequent inhibition of thrombin, reflect the three steps of coagulation: initiation, propagation, and termination [23]. Thrombin is the key enzyme within the coagulation process; coagulability can been defined as the capacity to generate thrombin and the enzymatic work that thrombin performs [24]. Thus, thrombin generation assay can check plasma coagulability [25]. In the present work, thrombin generation was assessed after plasma incubation for 30, 60, and 120 min upon contact with different materials to compare their coagulation potential. All parameters calculated for all materials from thrombin generation curves, did not reveal significant differences: *p* values are always >0.05.

Taken together, the results indicate that the hybrid membrane is compatible with blood.

## Limitations

One limitation of the present study is the use of a single donor’s blood. The absence of replicates is another limitation. However, both limitations are justified by the preliminary nature of the proposed investigation.

## Conclusions

The development of innovative TAHs to overcome the drawbacks of the currently available ones is still a challenging goal: it requires huge technological efforts (and financial investments as well) to design and create a device reproducing the performances of the native heart. Certainly, one critical issue is the hemocompatibility of blood-contacting surfaces. The present work allowed testing the blood-compatibility of a novel material (a hybrid membrane obtained by coupling a commercial polycarbonate urethane with decellularized porcine pericardium) just intended for the production of an innovative ventricular chamber. Platelet activation and thrombin generation were assessed upon human blood contact with the hybrid membrane, whose behavior was compared with other materials. Even though preliminary, this study demonstrated that the hybrid membrane possesses promising features to be usefully exploited for the production of blood-contacting components in mechanical circulatory supports.

## Glossary

ARLT: chronoflex AR-low tack
DPP: decellularized porcine pericardium
DPP-ARLT: Hybrid membrane obtained by coupling Decellularized Porcine Pericardium and Chronoflex AR-LT
NPPn: ative porcine pericardium
PNI: pseudo-neo intima
VAD: ventricular assist device
TAH: total artificial heart
TG: thrombin generation
